# Supplementary figures and images for: Autophagosome development and chloroplast segmentation occur synchronously for piecemeal degradation of chloroplasts
Source: eLife. 2024 Nov 7;12:RP93232. doi: 10.7554/eLife.93232 (PMC11542923; doi:10.7554/eLife.93232)

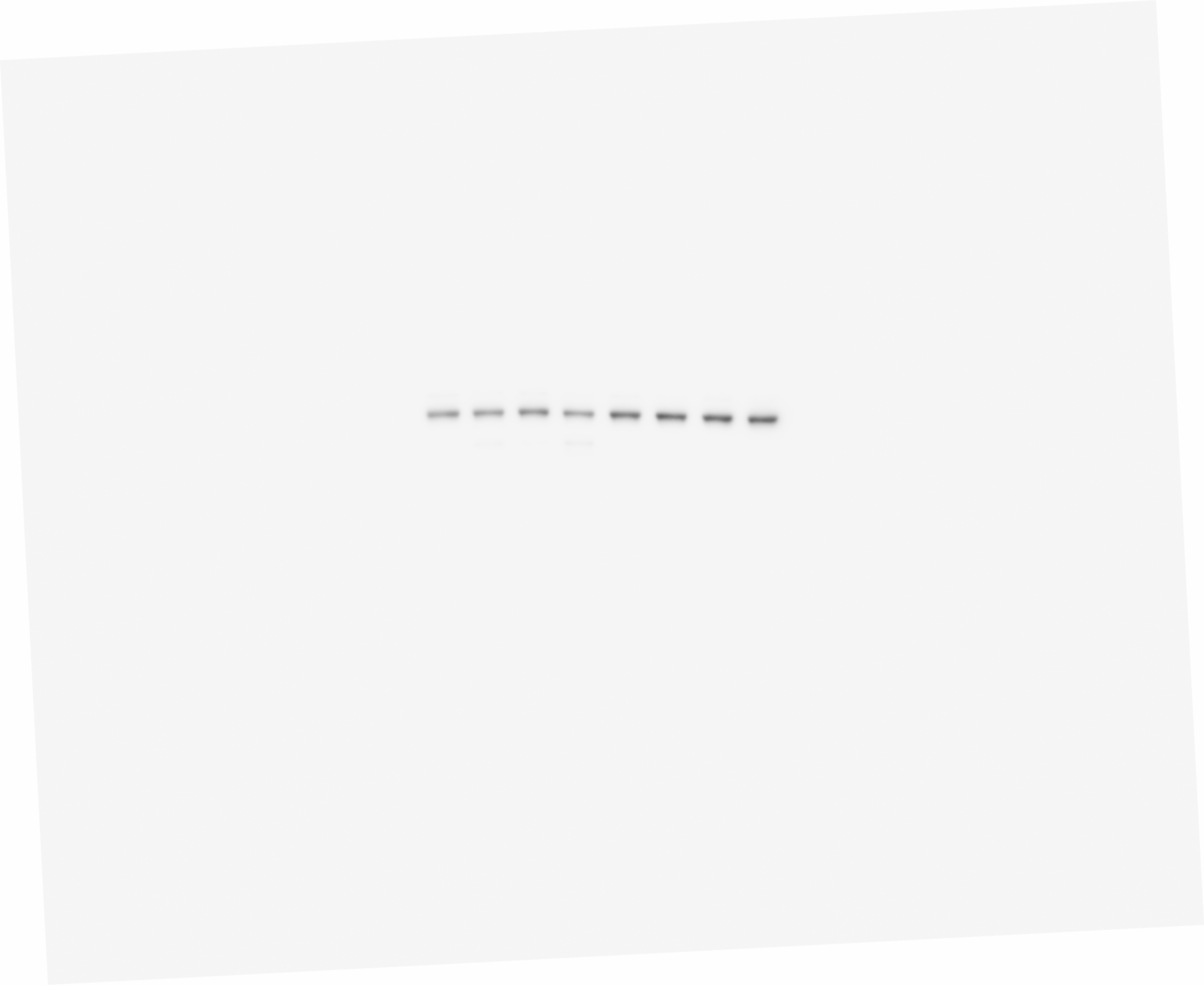

Supplement: Figure 8—source data 1. [file elife-93232-fig8-data1.zip › Figure_8-Source_Data_1/Figure_8C_anti-cFBPase.tif]

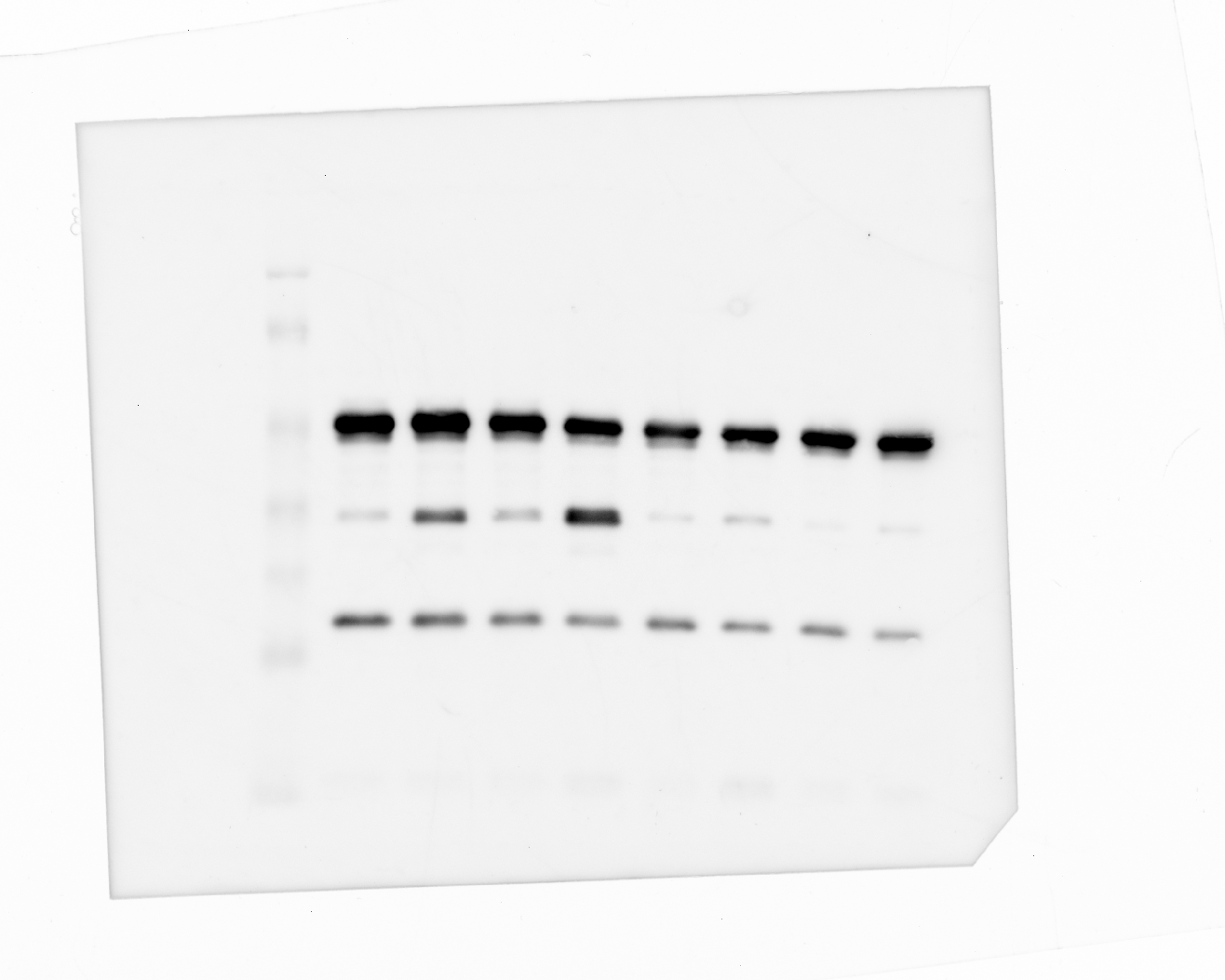

Supplement: Figure 8—source data 1. [file elife-93232-fig8-data1.zip › Figure_8-Source_Data_1/Figure_8C_anti-RFP.tif]

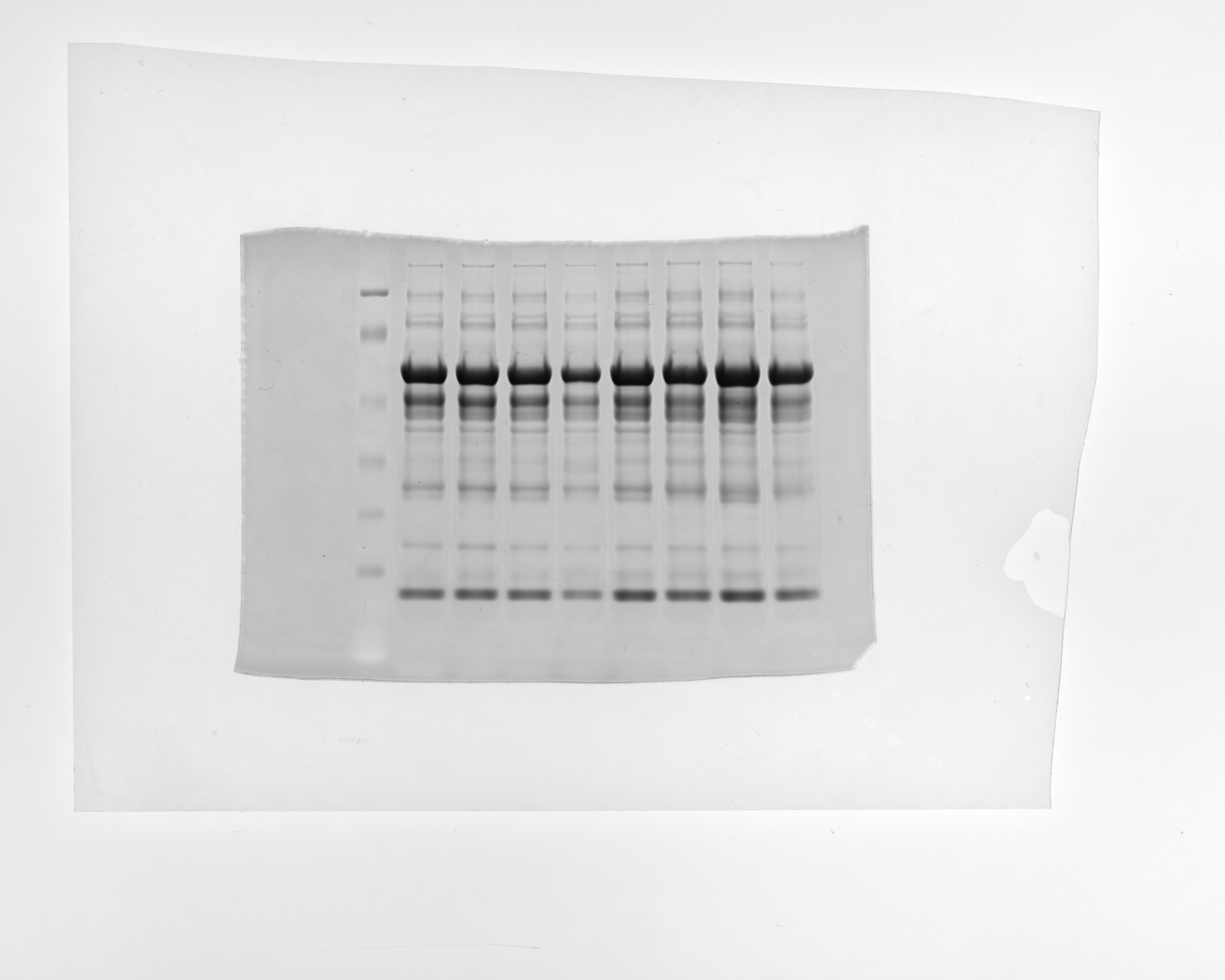

Supplement: Figure 8—source data 1. [file elife-93232-fig8-data1.zip › Figure_8-Source_Data_1/Figure_8C_CBBStain.tif]

Anti-RFP

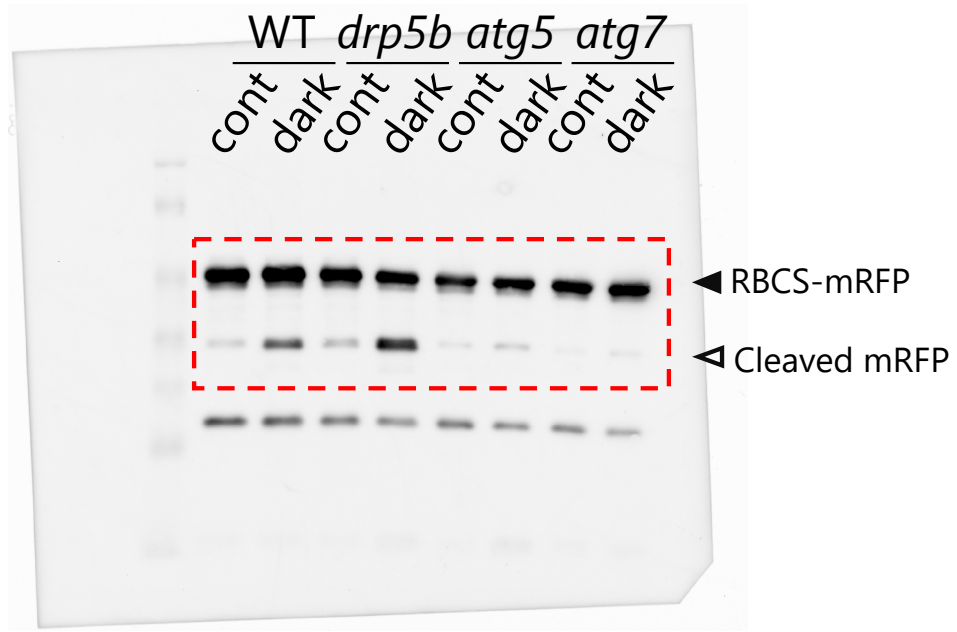

Anti-cFBPase

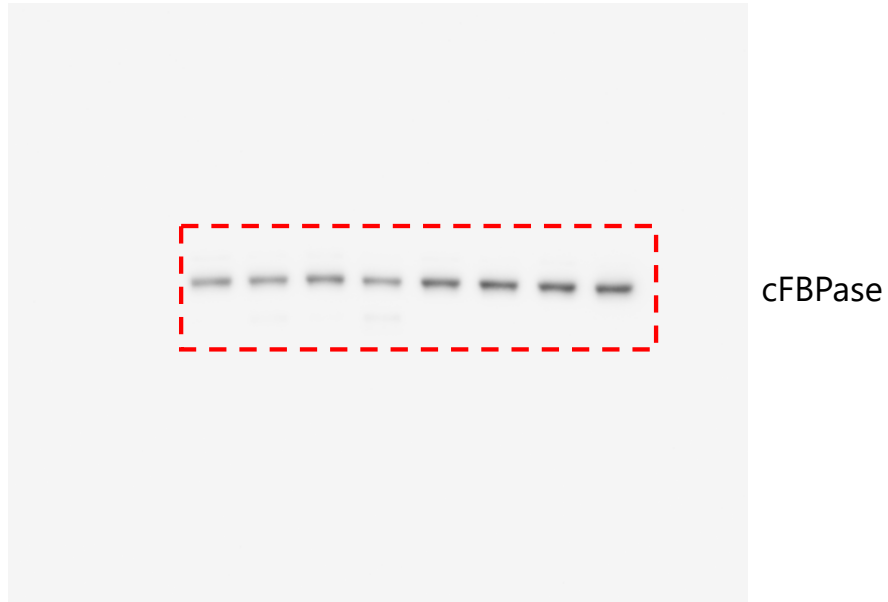

CBB stain

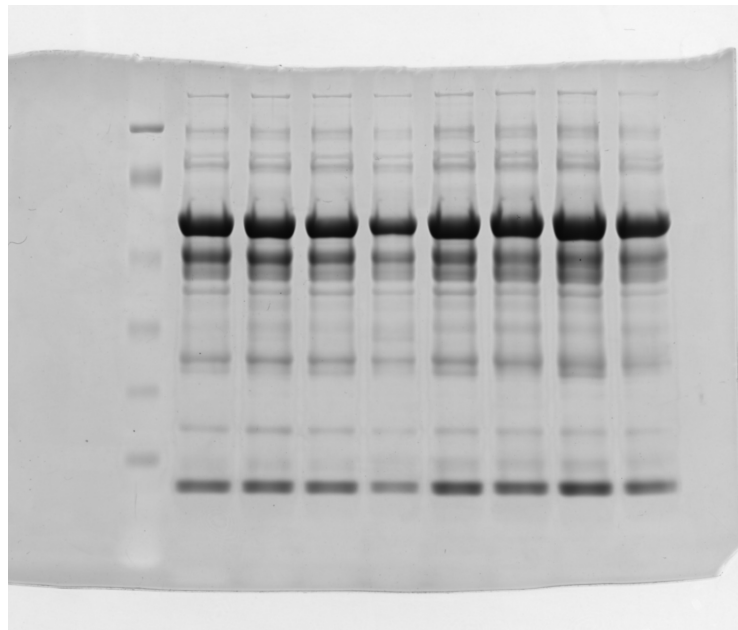

Supplement: Figure 8—source data 2. [file elife-93232-fig8-data2.zip › Figure_8-Source_Data_2.pdf]
